# Control of *Acinetobacter baumannii* outbreak in the neonatal intensive care unit in Latvia: whole-genome sequencing powered investigation and closure of the ward

**DOI:** 10.1186/s13756-019-0537-z

**Published:** 2019-05-22

**Authors:** A. Gramatniece, I. Silamikelis, Ie. Zahare, V. Urtans, Ir. Zahare, E. Dimina, M. Saule, A. Balode, I. Radovica-Spalvina, J. Klovins, D. Fridmanis, U. Dumpis

**Affiliations:** 1 Pauls Stradins Clinical University Hospital, Riga, Latvia; 2 University of Latvia, Riga, Latvia; 3 Latvian Biomedical Research and Study Center, Riga, Latvia; 4 Center for Disease Prevention and Control, Riga, Latvia

**Keywords:** *Acinetobacter*, *Baumannii*, Outbreak, Neonatal intensive care

## Abstract

**Background:**

*Acinetobacter baumannii* is an emerging pathogen capable of causing hospital-acquired infections (HAIs). It has the ability to survive on environmental surfaces for months, making transmission difficult to control. Our report describes the investigation and restriction of an outbreak of *A.baumannii* in the Neonatal Intensive Care Unit (NICU) using whole-genome sequencing (WGS) and multi-modal infection control measures.

**Methods:**

A prospective surveillance of HAIs was initiated in the NICU at the Pauls Stradins Clinical University Hospital (PSCUH) in Latvia on 1/9/2012 and identified an outbreak of *A.baumannii*. Case definitions for *A.baumannii* bloodstream infection (BSI) and colonization were implemented; surveillance cultures were obtained from all admitted patients to monitor the rate of colonization; an infection prevention and control team was formed and infection control interventions implemented. Environmental sampling of the NICU and Labour ward was performed. We employed WGS to differentiate phenotypically identical multidrug-resistant *A.baumannii* (MDRAB) strains from simultaneous intrahospital outbreaks in the adult Intensive Care Unit and NICU.

**Results:**

Between 1/9/2012 and 31/12/2017 the surveillance included 2157 neonates. A total of 17 neonates had *A.baumannii* BSI, with the highest rate of 30.0 cases per 1000 bed-days in November 2012. Rectal screening samples were positive for *A.baumannii-complex* in 182 neonates reaching 119.6 per 1000 bed-days in July 2015. All 298 environmental cultures were negative. Two phenotypically identical MDRAB isolates from the simultaneous intrahospital outbreaks were differentiated using WGS, ruling out an inter-ward transmission. Adherence to stringent infection control measures decreased BSI cases but colonization remained persistent. With several relapses, the outbreak was ongoing for four years. No new *A.baumannii* BSI cases were registered after total environmental decontamination in the NICU in July 2015. Colonization reappeared and persisted until in November 2016 when the ward was temporarily closed, relocated and renovated. No *A.baumannii* cases were registered after the renovation.

**Conclusion:**

The HAI surveillance system successfully detected and facilitated the control of the *A.baumannii* outbreak. Whole-genome sequencing was found to be a useful method for differentiation of phenotypically identical *A.baumannii* strains from the intrahospital outbreak. Only multi-modal infection control program, including closure, temporary relocation, and renovation of the ward, restricted the outbreak.

## Introduction

*Acinetobacter baumannii* has emerged worldwide as an important hospital-acquired infection (HAI) causing pathogen. [1, 2] The bacteria can survive in the environment for months, thus making transmission difficult to prevent and control. [3] *A.baumannii* can colonize the human skin and gastrointestinal tract and thereby can cause HAIs. [4–6] Neonates admitted to Neonatal Intensive Care Unit (NICU) are at increased risk of contracting HAIs due to their immature immune system and frequent invasive manipulations. [7–9] Bloodstream infections (BSIs) caused by *A.baumannii* occur primarily in premature low-birth-weight infants. [8] In 4-year surveillance of device-associated HAIs in a NICU in Turkey *A.baumannii* was described as the main cause of device-associated HAIs. [10] HAIs outbreaks are associated with higher mortality, morbidity and increased hospital costs. [11–13] Surveillance of HAIs is essential for detection of the outbreaks and their containment. [11, 14–20] Standard typing methods during the surveillance and investigation of the outbreak were found to be useful in revealing relationships between isolates but could not to resolve differences between closely related strains. Whole-genome sequencing has shown to be more sensitive in discriminating between closely related strains, including from intra-hospital outbreaks. [21–24]

In this report, we describe how an implementation of the HAIs surveillance system for the first time in a PSCUH NICU in Latvia led to the rapid detection of an outbreak of *A.baumannii*. The objective of this report is to summarize the investigation and restriction of a continuous outbreak using whole-genome sequencing and multi-modal infection control interventions.

## Methods

### Clinical setting

Pauls Stradins Clinical University Hospital (PSCUH) is an 860-bed teaching hospital providing primary and tertiary-care to adults and neonatal patients with approximately 47,700 admissions accounting for 255,000 patient-days per year. The Neonatal Intensive Care Unit (NICU) with seven intensive care beds is providing care to approximately 250 critically ill neonates yearly, all transferred from PSCUH Labour ward. The NICU is open-plan divided into two sections - three and four beds in each. Until September 2012 PSCUH NICU did not have a HAIs surveillance system. In September 2012 a prospective neonatal HAI surveillance system was adapted and introduced [25] in the NICU including all patients admitted from the PSCUH Labour ward to the NICU.

### Epidemiological data

Soon after the implementation of the HAIs surveillance system in September 2012, an *A.baumannii* outbreak was detected. A case definition of *A.baumannii* BSI was developed, using the definitions of the National Nosocomial Infections Surveillance Systems of the Centre for Disease Control and Prevention (CDC), adjusted for NICU patients. [25] Two months after the implementation of the surveillance system neonatal screening for *A.baumannii* colonization was initiated. Surveillance cultures of rectal swab specimens were obtained from all NICU patients at the admission and before the discharge. Colonization was defined as a rectal swab sample tested positive for *A.baumannii-complex*. Simultaneously samples from *A.baumannii* outbreak in the adult intensive care unit (ICU) with the phenotypically identical *A.baumannii* strain were collected. To resolve differences and exclude transmission between two wards situated in the same building, whole genome sequencing (WGS) was performed on matching *A.baumannii* samples isolated from adult ICU and NICU in the same outbreak period.

### Microbiology

Blood cultures were processed using the BacT/ALERT automated system (*bioMérieaux*). When the system alerted a positive result, gram staining was performed and inoculated on blood, MacConkey, and egg-yolk salt agars. After 24 h all lactose negative colonies were inoculated on Kligler iron agar, sulfur indole motility medium, and Simmons citrate agar. Anal swab specimens were inoculated on Levine agar and MacConkey agar. Further inoculations of lactose negative gram-negative pathogens were performed as previously described. All non-fermenting pathogens were identified using either Vitek GN, Vitek2 automated system (*bioMérieaux*) or BBL Crystal enteric/non-fermenter ID kit (Becton Dickinson) and reported as *A.baumannii-complex*. Antimicrobial susceptibility testing was performed using disk-diffusion testing as recommended by The Clinical & Laboratory Standards Institute (*CLSI*) for trimethoprim-sulfamethoxazole, amikacin, gentamicin, imipenem, ceftazidime, piperacillin-tazobactam, piperacillin, ampicillin-sulbactam.

### Whole-genome sequencing

Sequencing was carried out at the Latvian Biomedical Research and Study Centre using Life Technologies Ion Torrent™ PGM platform. [26] Invitrogen Qubit 2.0 Fluorometer (Invitrogen) was used to normalize genomic DNA concentration to 20 ng/μl. Standard barcoded Fragment library preparation protocol with DNA input of 1 μg and Ion Plus Fragment Library kit was used to prepare DNA libraries. Physical DNA fragmentation procedure was carried out using Covaris S220 focused-ultrasonicator (Covaris) with shearing parameters for 300 bp long DNA fragments: peak incident power – 175; duty factor - 10%; cycles per burst – 200; treatment time - 50s; temperature – 7 °C; water level S220–12; sample volume - 50 μl. Size selection was done using 1.5% Dye Free Agarose gel cassettes on Blue Pippin (Labgene scientific) automated DNA size selection and collection device; targeted elution of 390 bp long fragments were chosen. Commercially available, platform-specific barcodes were used during library preparation to distinguish different samples (Ion Xpress™ Barcode Adapters). The final library quantification and the quality check were performed on Agilent 2100 Bioanalyzer using High Sensitivity DNA chips (Agilent Technologies). The libraries were diluted to a concentration of approximately 19 pM and each of the six libraries were pooled together. Ion OneTouch™ 200 Template Kit v2 DL (Release: 12 September 2012, Publication No. MAN0006957) was used for template preparation and emulsion PCR. Ion PGM™ Sequencing 300 Kit (Release: 6 September 2012, Publication No. MAN0007062) and standard sequencing protocol for Ion 318™ chip type was used for the sequencing procedure. The predicted average coverage was ~30x.

### Whole-genome sequencing data analysis

The reference genomes were searched in NCBI’s GenBank using keywords ‘((Acinetobacter[Title]) AND “complete genome”[Title]) NOT plasmid[Title] NOT phage[Title] NOT bacteriophage[Title]’. As a result, 23 reference genomes were found from which 4 were excluded as duplicates. 200 bp reads were generated from retained reference genomes with the depth of coverage 100. Sequenced samples and generated reads were mapped against reference genome TCDC-AB0715 (acc. no.: NC_017387.1) using *tmap-3.4.1* mapping software. Mapping was performed using k-mer lookup algorithm (mapping procedure #3 in *tmap* software). PCR duplicates were removed using *Picard* and local realignment around indels was performed using *GATK-3.1-1.* SNP calling was performed using GATK’s UnifiedGenotyper SNP calling an algorithm. Further SNP filtering was applied with custom *Python* script retaining only SNPs with B-allele frequency at least 0.6 and depth of coverage at least 10 (statistics were calculated from VCF file using fields “AD” and “DP” respectively). Resulting SNPs were concatenated, creating genotypes for each sample. Phylogenetic analysis on resulting genotypes was performed using *MEGA 5* software with the UPGMA algorithm and bootstrap on 500 replicates.

### Infection control interventions

After identification of the *A.baumannii* outbreak in September 2012, an Infection Prevention and Control (IPC) team was formed in PSCUH from the local staff available (one IPC nurse, one Infectious Disease (ID) specialist, one junior doctor, one epidemiologist). The team visited NICU weekly reporting every new *A.baumannii* case back to the ward staff. Multi-modal infection control program was introduced: recommendations for patient and ward area improvement according to IPC principles were developed; hand hygiene procedures were reassessed and intensified; adherence to infection control measures was controlled by the IPC team; meetings with the hospital board were held; invasive manipulation indications and procedures were reviewed; patient screening for *A.baumannii* colonization at the admission and before the discharge was initiated; parenteral and enteral feeding guidelines and procedures were revised and re-implemented; environmental screening and decontamination was performed repeatedly. (Fig. 2).

### Environmental investigation

Environmental cultures were collected from multiple high-touch surfaces in the NICU (e.g. computer keyboard, desks, patient monitors), medical devices (e.g. ultrasonography device, intubation equipment), feeding equipment (e.g. milk pumps, bottles, feeding mixtures), hands of the healthcare workers and environment in the ward (e.g. room air, tap aerators, water, clothing and sheets, disinfectant containers). The ventilation and air conditioning system was assessed, disassembled, revised and cultured. Extensive environmental sampling was performed in the Labour ward to exclude *A.baumannii* transmission considering that all neonates are admitted to the NICU from PSCUH Labour facilities.

## Results

### Outbreak description

**Fig. 1:**
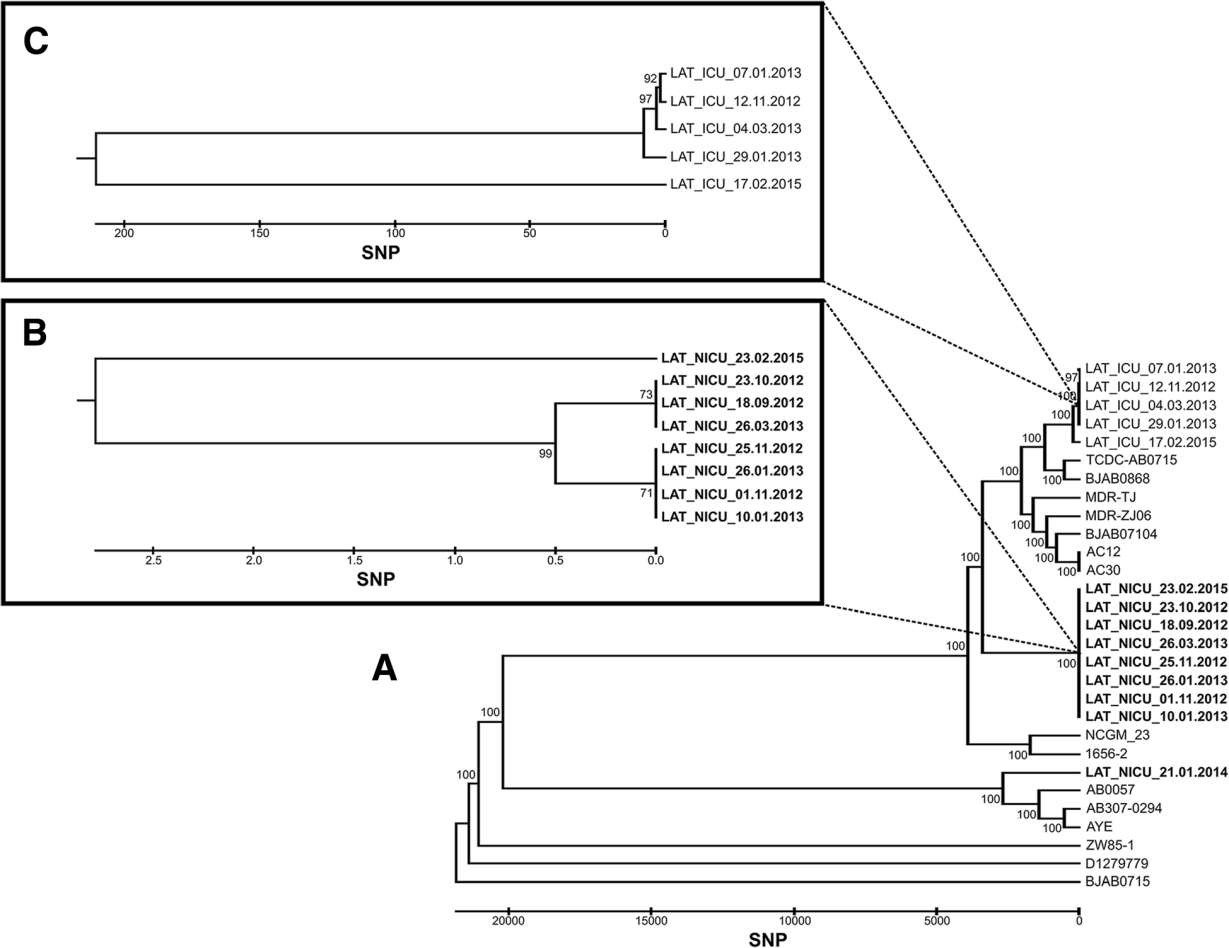
Phylogenetic tree of *A.baumannii* strains isolated in adult ICU (LAT_ICU_dd.mm.yyyy., panel **c**), NICU (LAT_NICU_dd.mm.yyyy., panel **b**) and LAT_NICU_21.01.2014. strain (**a**)

In the surveillance, we included 2157 neonates admitted to PSCUH NICU from 1/9/2012 until 31/12/2017. In September 2012, because 13.3 *A.baumannii* BSI cases per 1000 patient days were detected, we initiated an outbreak investigation and implemented a multi-modal infection control program. From 1/9/2012 until 31/12/2017 applying case definitions we identified 17 *A.baumannii* BSIs, 70.6% of infants had very low birth weight (< 1500 g according to the World Health Organization definition). In patients with BSIs mean birth weight was 1244.6 g ± 732.2 g, mean gestational age was 27.5 ± 2.1 weeks, 53% of the patients were male. Two patients with *A.baumannii* BSI died during the surveillance period. The highest incidence of *A.baumannii* BSIs was registered in November 2012 reaching 30.0 cases per 1000 patient days. From 1/11/2012, when colonization screening was initiated, until 31/12/2017 we identified 182 *A.baumannii* colonization cases. The highest colonization rate was in July 2015 reaching 119.6 cases per 1000 patient days. An outbreak with several relapses was ongoing for four years. All patient rectal samples obtained before the admission in the NICU were negative for *A.baumannii-complex*. All *A.baumannii* isolates in the NICU had the same resistance pattern being resistant to trimethoprim/sulfamethoxazole, piperacillin, and gentamicin, except one *A.baumannii* isolate (Fig. 1 LAT_NICU_21.01.2014.) resistant to imipenem, piperacillin/tazobactam, ceftazidime, trimethoprim/sulfamethoxazole. This strain was phenotypically identical to the strains from the simultaneous multidrug-resistant *A.baumannii* (MDRAB) outbreak in the adult ICU located in the same hospital building.

### Infection control interventions

**Fig. 2:**
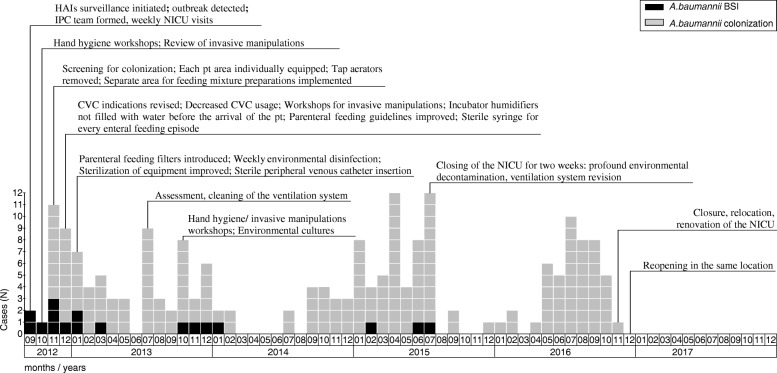
*A.baumannii* BSI and colonization cases in relation to infection control interventions. *BSI* bloodstream infection, *CVC* central vascular catheter, *N* number, *NICU* Neonatal Intensive Care Unit, *HAI* hospital-acquired infection, *IPC* infection prevention and control, *pt.* patient

In September 2012, after the detection of the outbreak, we did environmental assessment in the NICU and developed recommendations for patient and ward area improvement according to the IPC principles. (Fig. 2) The IPC team controlled adherence to the infection control measures during regular meetings with the NICU staff.

In October 2012 hand hygiene was intensified and educational workshops held for doctors and nurses; invasive procedures (intubation, central vascular catheterization (CVC), feeding with a nasogastric tube, parenteral feeding) were reviewed.

In November 2012 *A.baumannii* BSIs incidence was registered to reach 30.0 cases per 1000 bed-days. Rectal swab screenings for *A.baumannii* colonization at the admission and before the discharge were initiated; desks and medical documentation were relocated from the patient area; each patient’s zone was equipped with individual equipment; room for feeding mixture and milk preparation with clean and potentially contaminated areas was allocated; tap aerators were removed and cultured. The infection control nurse participated in invasive manipulations and educated NICU staff and patients’ family about the IPC procedures.

During December 2012 numerous workshops about invasive manipulations were held; central venous catheterization and umbilical catheterization indications were revised; the use of CVC was decreased; incubator humidifiers were not filled with water before the arrival of the patient anymore. The parenteral feeding mixture preparation guidelines were revised; the enteral feeding procedure was changed – sterile syringe was used before every new feeding episode.

In January 2013 parenteral feeding filters were introduced in the ward; weekly environmental disinfection in the NICU was initiated; the process of sterilization of reusable equipment was re-evaluated and improved; sterile peripheral venous catheter insertion was implemented. As infection control interventions were intensified *A.baumannii* rates decreased. From April 2013 until October 2013 no new *A.baumannii* BSIs were registered. In addition, *A.baumannii* colonization cases decreased until no cases were registered in June 2013.

In July 2013 during the summer heat, *A.baumannii* colonization peaked again reaching 52.0 cases per 1000 bed-days. After the assessment of the risk factors, we identified that the increase of colonization cases was after the air conditioning system was turned on. Together with the Technical Department the ventilation system was assessed and cultured. All cultures came back negative for *A.baumannii*.

In October 2013 another case of *A.baumannii* BSI was detected after a six-month break. Re-assessment of the invasive manipulations and workshops of hand hygiene were re-implemented, the environment was recultured and meetings with the Hospital Board were held. Registered cases decreased due to intensified infection control measures. No new *A.baumannii* BSI cases were registered for more than a year from February 2014 until January 2015. But after the peak of colonization in January 2015 we detected a BSI in February 2015. Two more BSI cases were identified in June and July 2015. Nevertheless, the highest rate of *A.baumannii* colonization was detected in July 2015 reaching 119.6 cases per 1000 patient-days. The decision to close the NICU for two weeks for profound environmental decontamination and ventilation system revision was made. Complete closure with the renovation was not possible that time due to financial constraints. Since reopening and stricter infection control measures in the NICU, no new *A.baumannii* BSI cases were registered. Strengthened infection control interventions restricted *A.baumannii* BSI cases, but colonization re-emerged indicating that environmental source remained undetected.

In May 2016 colonization rates increased repeatedly reaching 89.0 cases per 1000 bed-days in July 2016 when air condition was used. The infection control activities were intensified again. Repeated meetings with the Hospital Board were held insisting on the closure of the NICU for ventilation system repair and renovation. In November 2016, the decision to close the ward was made. NICU was temporarily relocated, the ventilation system was disassembled, re-cultured and repaired. The renovation of the ward was ongoing for three weeks. During the relocation and after reopening of the ward in the same location no new *A.baumannii* cases were registered.

### Environmental sampling

In total 298 environmental cultures were collected with extensive sampling on high-touch surfaces in NICU and Labour ward and were all negative for *A.baumannii*.

After the assessment of the risk factors, the ventilation system seemed to be the possible source of the outbreak. Thus together with the Technical Department, we performed a profound examination and complete disassembly of it. We identified that the ventilation system had numerous construction failures and it had not been cleaned nor examined. Also, periodically there were inappropriate room temperatures due to a malfunction of the air conditioner. The ventilation system was cleaned, disinfected, repaired and cultured repeatedly for *A.baumannii–* all cultures were negative.

### Whole-genome sequencing

All *A. baumannii* isolates from the NICU had the same antimicrobial resistance pattern and were phenotypically identical, except one MDRAB causing BSI (Fig. 1 LAT_NICU_21.01.2014.) resistant to imipenem, piperacillin/tazobactam, ceftazidime, trimethoprim/sulfamethoxazole. This strain was phenotypically identical to the strains isolated from simultaneous MDRAB outbreak in PSCUH adult ICU. Both wards are located in the same building so we suspected inter-ward transmission of the bacteria. To resolve the differences between these closely related strains, we performed WGS. It was carried out on five samples of MDRAB from the adult ICU matching retrospectively nine samples from the NICU (LAT_ICU_dd.mm.yyyy.) outbreak – two samples from each year (2012, 2013, 2015) and a sample from 21/01/2014, identical to the strain from adult ICU. As the phenotypical pattern of the selected samples from the outbreak period suggested a single strain outbreak, except isolate LAT_NICU_21.01.2014, WGS was not performed to all *A.baumannii* isolates from NICU. The phylogenetic analysis of WGS identified three distinct clones. NICU isolates clustered together, while LAT_NICU_21.01.2014. phylogenetically was distinct from all other strains isolated from both - NICU and adult ICU - during the same period. Thus WGS excluded inter-ward transmission (Fig. 1). The MDRAB strain from NICU did not spread in the ward and was apparently contained by the intensified infection control measures.

## Discussion

Here we report a rapid identification of an outbreak of *A. baumannii* resulting from an HAI surveillance system implementation for the first time in PSCUH NICU. We successfully used WGS to characterize an epidemiological pattern of the intrahospital outbreak of two phenotypically identical *A.baumannii* strains. The formation of the IPC team and implementation of the multi-modal infection control program, including renovation of the ward, were the key elements to contain the outbreak.

Previously published studies have shown that infection control measures are instrumental in the containment of the HAIs outbreaks. [27, 28] As soon as our outbreak was detected, the IPC team from the staff available at our hospital was formed and respective infection control interventions were intensified. We employed various infection control activities such as hand hygiene workshops, IPC education for the staff and patients families, environmental cleaning, etc. These interventions seemed to be effective only in reducing *A.baumannii* BSI cases. Despite all the effort colonization cases re-emerged suggesting an environmental source. (Fig. 2).

Restriction of the spread of HAIs in NICU is challenging, especially if the outbreak is caused by *A.baumannii* because the bacteria can survive in the environment for long periods of time. [3] In some studies, investigators have managed to isolate the outbreak-causing pathogen from hygroscopic bandages [11], ventilator surfaces, bedside curtains, and a bed rail [29], but most of the studies have failed to identify the source. [28, 30] Even in the large scale *Serratia marcescens* outbreak in the NICU in Germany more than 600 environmental samples were taken and all were found to be negative. [31] We failed to identify the source after taking almost 300 environmental samples, though the pattern of the epidemic curve (Fig. 2) strongly indicated possible environmental contamination followed by further spread through contact transmission. Culture-based methods might not be sensitive enough to identify low-level environmental contamination. Real-time polymerase chain reaction (R-T PCR) assay has demonstrated better sensitivity. [32] Though the presence of the genomic DNA of *A.baumannii* could be associated with the identification of nonviable remains of the bacteria being present after decontamination, it could be helpful to identify the source. At that time R-T PCR assay was not available in our setting. An alternative explanation of the negative cultures in the NICU could be the reintroduction of bacteria from an outside source but no evidence to support this was found. All environmental samples from the Labour ward and rectal samples from patients before the admission were also negative. Thus, we were not able to confirm environmental contamination with positive cultures, even when the epidemiological curve (Fig. 2) suggested it.

After the assessment of the risk factors, we noticed that the colonization rate increased in the summer heat when the air conditioner was turned on. Significant shortcomings of the ventilation system were identified after assessment of the ward together with the Technical Department. The ventilation system was cleaned and cultured repeatedly. All cultures from the room air and ventilation were negative. Lack of finances led to delays in the renovation of the ward including complete disassembly and repair of the ventilation system. The decision on closure of the ward for two weeks with profound cleaning and culturing of the ventilation system was made. This strategy seemed to contain the *A.baumannii* BSIs and no new cases were detected since August 2015. Profound cleaning of the ward was described as a strategy in the control of *A.baumannii* in the previous study by Munier et al. [33] However, further colonization cases were identified even after this intervention. (Fig. 2).

Colonization persisted and we still could not identify the point source. Since the general characteristics of the patients, including birth weight and gestational age, were similar over the surveillance period, we assume that those did not significantly affect the BSI and colonization rates. The source seemed to be the ventilation system despite the negative culturing results. Finally, the decision to close the ward for the renovation including repair of the ventilation system was made. Temporary closure, relocation, and renovation of the ward managed to eliminated cases of colonization. We believe that the unidentified environmental source was eradicated.

During an outbreak, it was important to characterize the genetic relationship between isolates. Phylogenetic relationship of the strains could be critical in guiding the infection control measures and restricting the outbreak. Whole-genome sequencing technology has been recognized to be more effective than the traditional methods for characterization of the outbreaks caused by Gram-negative bacteria. [34, 35] This was the first time WGS powered investigation was used in Latvia to resolve the intrahospital outbreak of *A.baumannii*. The phylogenetic analysis excluded inter-ward transmission between the adult ICU and NICU. A phenotypically identical but phylogenetically different MDRAB strain (LAT-NICU-21.01.2014 in Fig. 1) was identified in the NICU and was apparently contained by the infection control measures. This could also emphasize environmental contamination by the predominant strain.

## Conclusions

We have described an outbreak in the NICU caused by *A. baumannii* contained with a multi-modal infection control program, including closure, temporary relocation, and renovation of the ward. Introduction of the HAI surveillance system was a crucial step towards timely identification and control of the outbreak. Whole-genome sequencing was found to be a useful method for tracking *A.baumannii* and differentiated phenotypically identical strains. We conclude that only multi-modal infection control interventions contained the outbreak and could be recommended in similar occasions.

## Abbreviations

BSI: Bloodstream infection
CDC: Center for Disease Prevention and Control
CLSI: Clinical & Laboratory Standards Institute
CPAP: Continuous positive airway pressure
CVC: Central vascular catheters
EUCAST: The European Committee on Antimicrobial Susceptibility Testing
*g*: *grams*
HAI: Hospital-acquired infection
ICU: Intensive care unit
ID: Infectious disease
IPC: Infection prevention and control
MDRAB: Multidrug-resistant *A.baumannii*
N: Number
NICU: Neonatal intensive care unit
PSCUH: Pauls Stradins Clinical University Hospital
pt.: Patient
WGS: Whole-genome sequencing
